# Hypoglycemic activity of the ethyl acetate extract from *Smilax glabra* Roxb in mice: Biochemical and histopathological studies

**DOI:** 10.22038/ijbms.2020.46658.10763

**Published:** 2020-12

**Authors:** Phuong Thi Mai Nguyen, Quang Van Ngo, Minh Thi Hong Nguyen, Lien Thi Quach, Stephen G Pyne

**Affiliations:** 1 Institute of Biotechnology, Vietnam Academy of Science and Technology, Hanoi, Vietnam.; 2 Graduate University of Science and Technology, Vietnam Academy of Science and Technology, Hanoi, Vietnam; 3 Institute of Chemistry, Vietnam Academy of Science and Technology, Hanoi, Vietnam; 4 University of Science and Technology of Hanoi, Vietnam Academy of Science and Technology, Hanoi, Vietnam; 5 School of Chemistry and Molecular Bioscience, University of Wollongong, Wollongong, New South Wales, Australia

**Keywords:** Alpha-glucosidase, Alpha-amylase, Diabetes, Ethyl acetate (EtOAc) – extract, In vivo model, Smilax glabra Roxb

## Abstract

**Objective(s):**

This research was carried out to investigate the hypoglycemic activity of the ethyl acetate (EtOAc) extract from the roots of *Smilax glabra* Roxb, which strongly exhibit inhibitory activity against α-glucosidase and α-amylase on *in vivo* type 2 diabetic model.

**Materials and Methods:**

Column chromatography combined with crystallization was used to isolate the active fraction and compounds. Chemical structures of the compounds were determined based on the analysis of the spectroscopic data and comparison with the literature data. The α-glucosidase inhibitory activity (AGI) and the α-amylase inhibitory activity (AAI) were determined quantitatively spectrophotometrically using p-nitrophenyl α-D-glucopyranoside and soluble starch as substrates, respectively. The hypoglycemic activity was examined by evaluating its effects on glucose and insulin levels, insulin resistance, and histopathology of the pancreatic islets and livers in diabetic induced mice administrated with nicotinamide-streptozotocin.

**Results:**

The EtOAc extract and the bioactive compounds astilbin and 5-O-caffeoylshikimic acid in the extract were isolated and confirmed in structures, AGI, and AAI. The treatment at the doses of 500 and 1000 µg/kg of body weight reduced blood glucose levels down to the physiological level of the physical controls in the diabetic mice after two weeks (*P*<0.05). Moreover, the treatment improved insulin sensitivity. Histopathology analysis showed recovering effects in the size of the pancreatic islets and no damaging effects on the liver after treatment compared with the control group.

**Conclusion:**

Our data suggest that the EtOAc extract possesses hypoglycemic activity and has an antidiabetic potential for therapeutic applications.

## Introduction

Diabetes is a metabolic disease caused by inadequate insulin action, resulting in high blood glucose diabetes, especially noninsulin-dependent diabetes mellitus (type 2 diabetes) characterized by postprandial hyperglycemia is a global health affecting about 90% of the patients (1). Therapeutic approaches to decrease postprandial hyperglycemia include: i) inhibiting the activity of carbohydrate hydrolyzing enzymes, such as α-glucosidase (AGO) and α-amylase (AA), which help to reduce glucose levels going into the blood; ii) increasing insulin production in beta cells; and iii) enhancing insulin sensitivity of cells in tissues (2-6).

Treatment using synthetic drugs is not the best choice because of inefficacy and side effect problems. Plants are an abundant source of natural compounds that are potential antidiabetic agents with little or no side effects (2,7-9). Therefore, natural products from plants are promising alternatives for the control of hyperglycemia. 

*Smilax glabra *Roxb is a popular medicinal plant in Asian countries for the treatment of various chronic diseases (10). However, its hypoglycemic activity has not been intensively investigated. Our previous report (11) indicated that the EtOAc extract of this plant possesses high inhibitory activity against both AGO and AA with IC_50 _values of about 1 µg/ml and 120 µg/ml, respectively. These inhibitory activities were much higher than those of the single compounds, astilbin and 5-*O*-caffeoylshikimic acid, suggesting that the extract activity has synergistic effects from its constituents. The additive effects of the crude extract from this plant were also found by other researchers (12). This study was carried out to confirm the antidiabetic activity of the EtOAc extract from *S. glabra *on *in vivo* model via oral route by evaluating its effects on glucose and insulin levels, insulin resistance, histopathology of the pancreatic islets, and livers in type 2 diabetic mice.

## Materials and Methods

***Plant material***

The roots of *S. glabra *Roxb were collected in Thai Nguyen province in September 2016 and identified by Prof Phan Ke Loc. The voucher specimen (SGR92016) was deposited at the Institute of Ecology and Biological Resource, Vietnam Academy of Science and Technology, Hanoi, Vietnam.

***Isolation of the active fraction EtOAc and main active compounds in the extract***

The fine powder of *S. glabra* roots was macerated with 96% ethanol (EtOH) at room temperature for 72 hr (3 times) and then the solvent was removed under reduced pressure to obtain the EtOH residue. The residue was suspended in H_2_O, partitioned with n-Hexane (3 times) and then EtOAc (3 times). The EtOAc fraction was concentrated under reduced pressure to yield the EtOAc extract for isolation of desired compounds as described in a previous study (11). 

Isolation of the main bioactive compounds from EtOAc extract was done as previously described (11). Briefly, the EtOAc extract was subjected to column chromatography on silica gel using a solvent system of dichloromethane/methanol (MeOH): 8/2, (v/v) to obtain nine subfractions (F1 - F9). The first major active compound astilbin was obtained from fraction F6 by crystallization and then was further purified by recrystallization in MeOH/H_2_O (1/1, v/v). The second active compound 5-*O*-caffeoylshikimic acid was isolated from fraction F7 using C18 reversed-phase CC (acetone/H_2_O: 3/1, v/v). The chemical structures of the two of these compounds were confirmed by ^1^H and ^13^C NMR analysis combined with the references.

***Liquid chromatography – Mass spectrometry (LC-MS)***

Liquid Chromatography (LC) was performed on the EtOAc fraction from *S. glabra *as described previously (11) to confirm the presence of the two active compounds astilbin and 5-*O*-caffeoylshikimic acid 

***Assay for α-glucosidase inhibitory activity (AGI)***

AGI was determined according to the method described previously (11). The enzyme was incubated with the samples for 5 min before adding the substrate that was prepared in water. The inhibition was measured spectrophotometrically in 20 mM sodium phosphate buffer pH 6.0 at 37 °C using 3.0 mM *p*-nitrophenyl α-D-glucopyranoside (Sigma) as a substrate and 0.25 units/ml from *Saccharomyces cerevisiae* (Sigma). Acarbose (1.0 mM) was used as a positive control (BioTek ELx808 microplate reader, USA).

***Assay for amylase inhibitory activity (AAI)***

AAI was measured using starch–iodine assay in a microplate (13). Assay reactions were initiated by adding 40 µl of starch (Sigma S-2630) solution (2.0 g/l) and 40 µl of the enzyme in 0.1 M phosphate buffer at pH 7.0 to the wells. The enzyme was incubated with the samples for 5 min before adding the substrate (soluble starch) which was prepared in water. After 30 min of incubation at 37 °C, 20 µl of 1M HCl was added to stop the reaction, followed by the addition of 100 µl of iodine reagent (5 mM I_2_ and 5 mM KI). The absorbance at 580 nm was measured using a microplate reader (Bio-TEK EL x 808 microplate reader, USA).

***Diabetic induced mouse model using nicotinamide-streptozomycine (type 2 diabetes)***

White Swiss mice, provided by the animal house at the Army Medical Institute, were stabilized for 3 days then fasted overnight. Type 2 diabetes was established by the method described previously (14). Briefly, mice were fasted overnight. In the next morning, mice were injected intraperitoneally with nicotinamide (NA, Sigma) at a dose of 120 mg/kg (soluble in physiological saline), then after 15 min, they were injected intraperitoneally with streptozotocin (STZ, Sigma) at a dose of 50 mg/kg (dissolved in citrate buffer, pH 4.5). The samples were made freshly just before injection. After 72 hr of STZ-NA injection, blood glucose was qualified using a glucose kit (Erba, US). Mice with blood glucose levels higher than 200 mg/dL were considered diabetic mice (type 2) and were used in the study. The 50 qualified mice were divided into 5 groups, including: 

- Control group: physiological mice, no diabetes

- Diabetic group: diabetic mice 

- Metformin group: diabetic mice treated with metformin

at a dose of 200 mg/kg of body weight for 2 weeks. 

- Treatment 500 µg/kg group: diabetic mice treated with the extract at a dose of 500 µg/kg of body weight for 2 weeks.

- Treatment 1000 µg/kg group: diabetic mice treated with metformin at a dose of 1000 µg/kg of body weight for 2 weeks.

After 2 weeks of treatment, blood glucose and insulin levels were measured using commercial kits. The indexes to assess insulin resistance were then calculated, including HOMA-IR (homeostatic model assessment of insulin-resistance); HOMA-β (homeostatic model assessment of pancreatic β-cell function); QUICKI (quantitative insulin sensitivity check index); DI (insulin disposition index):

HOMA-IR = blood glucose (mg/dL) × blood insulin (µIU/ml)/405

HOMA-β = 20 × blood insulin (µIU/ml)/ (blood glucose (mMol/l) - 3.5)

QUICKI = 1/ (log blood glucose (mg/dl) + log blood insulin (µIU/ml) 

DI = HOMA-β/HOMA-IR 

***Histological assessment***

After taking blood for glucose and insulin tests, mice surgery was quickly performed for evaluation of pancreatic weight changes (% compared with body weight) and histopathology analysis. The livers and pancreases were immediately fixed in a 10% formalin solution then stained with HE (hematoxylin & eosin). Six stained pancreatic specimens were prepared for each mouse with 6 µm thick sections. The changes in the size of the pancreatic islets and liver damage (if found) were then evaluated. The diameter of the pancreatic islets was measured using ImageJ software. This experiment was performed at the Forensic Anatomy Department – 103 Hospital in Hanoi, Vietnam.

***Statistical analysis***

Statistical comparison between different groups was performed using one-way analysis of variance (ANOVA) and Tukey HSD test with *P*<0.05. Data are expressed as means±SE.

## Results

***S. glabra extract***

The EtOAc extract of *S. glabra* for *in vivo* experiments in this study was prepared as described in the materials and methods section and was determined for AGI and AAI activity. Data in Table 1 indicate that the EtOAc extract exhibited AGI and AAI activity with IC_50_ values of 1.15 µg/ml and 133 µg/ml, respectively. Interestingly, these IC_50_ values of AGI activity are significantly lower than that of acarbose (1.15 µg/ml vs 359.21 µg/ml).

**Figure 1:**
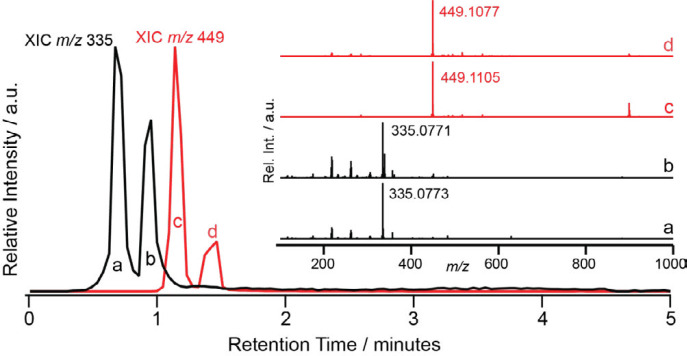
Extracted ion chromatogram (XIC) for *m/z* 335 in black and 449 in red from LC-MS of ethyl acetate extract from *Smilax glabra*. The inset shows accurate mass spectra resulting from averaging under each of the corresponding peaks in the chromatograms

 LC-MS was performed in an attempt to better separate and analyze online the components of the EtOAc extract. Figure 1 shows the extracted ion chromatograms (XICs) for the *m/z* channels that would carry deprotonated astilbin (red trace, *m/z* 449) and 5-*O*-caffeoylshikimic acid (black trace, *m/z* 335). Accurate mass spectral features from the two major peaks in each XIC give errors within 3.5 and 0.5 ppm for [C_21_H_22_O_11_ – H]^-^ and [C_16_H_16_O_8_ – H]^-^, respectively, supporting the NMR structural assignments of astilbin and 5-*O*-caffeoylshikimic acid. Interestingly, the two nearly baseline resolved features in each XIC suggest the presence of isomers under each of the targeted *m/z* channels. Thus, LC-MS data indicated that the EtOAc extract contains active compounds astilbin and 5-*O*-caffeoylshikimic acid as found in our previous study (11).

***Isolation of astilbin and 5-O-caffeoylshikimic acid from the EtOAc extract***

**Figure 2:**
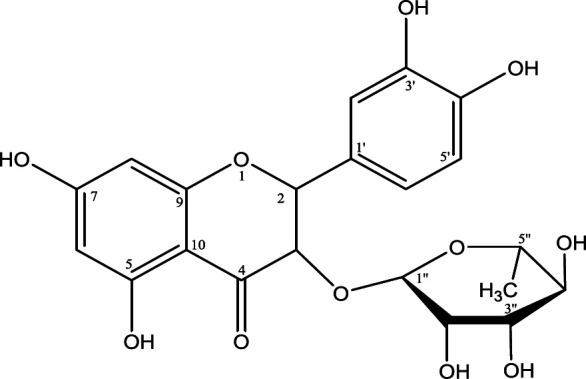
Structure of astilbin from *Smilax glabra* (C_21_H_22_O_11_; MW=450)

**Figure 3:**
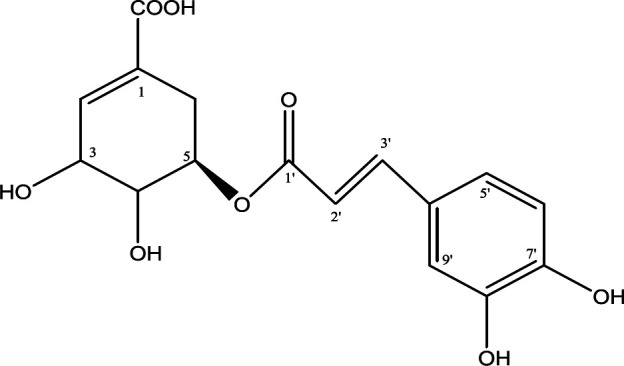
**S**tructure of 5-O-caffeoylshikimic acid from *Smilax glabra* (C_16_H_16_O_8_; MW=336)

The chemical structures of the two major active compounds in the EtOAc extract, to be used for testing on the *in vivo* model, was confirmed by ^1^H and ^13^C NMR analysis combined with references. The data interpretation of the two compounds (Table SI and Table SII) compared with the references indicated they are astilbin (Figure 2) and 5-*O*-caffeoylshikimic acid (Figure 3). The two compounds were determined as major constituents that possess AGI (11). Especially, 5-*O*-caffeoylshikimic acid was for the first time found exhibiting AGI in our study (11). The IC_50_ values of AGI and AAI of these two compounds were 135 µg/ml and 1.2 mg/ml for astilbin and 46 µg/ml and 0.8 mg/ml for 5-*O*-caffeoylshikimic acid, respectively. The IC_50_ values for AGI of the two compounds are about 3 and 8 folds lower than that of acarbose (359.21 µg/ml) (data not shown).

***Effects of the EtOAc extract on glucose and insulin levels in the blood***

The data in Table 2 indicate that after two weeks of treatment, the blood glucose level in the model group (diabetes without treatment) was still significantly higher than that of the control and the sample treatment groups (*P*<0.001). However, the insulin level in the model group was slightly decreased but was not significantly different from that of the control and treatment groups (*P>*0.05). Interestingly, metformin and EtOAc extract clearly reduced blood glucose levels in diabetic mice (*P*<0.001) but there was no change in the insulin levels. Metformin at a dose of 200 mg/kg and EtOAc extract at a dose of 1000 mg/kg of body weight reduced blood glucose levels down to that of the control group (*P*<0.05). Although the reduction of blood glucose was clearly observed in the treatment group at a dose of 500 mg/kg of body weight, it was still higher than that of the control group (*P*<0.05).

***Effects of the EtOAc extract on insulin resistance***

Our obtained data here also showed that the EtOAc extract can reduce STZ-NA induced problems by lowering blood glucose levels and improving indexes related to insulin resistance, including HOMA-IR, HOMA-β, QUICKI, and DI without changing insulin levels. These indices are determined to assess the function of insulin-producing cells and the action of insulin (15-17). HOMA-IR is used to quantify insulin resistance and HOMA-β is used to quantify cellular function. Here, at medium and high doses of treatment, the extract had the effects of restoring the health of pancreatic cells and restoring insulin sensitivity by reducing the HOMA-IR index, increasing HOMA-β, DI and QUICKI to the same level as that of the control group. The high treatment dose (1000 mg/kg of body weight) showed better effects than the lower dose (500 mg/kg of body weight). 

The data presented in Table 3 show that the treatment group at a dose of 500 mg/kg of body weight had HOMA-IR and QUICKI indexes recovered back to the level of the control group (*P*>0.05), while the HOMA-β and DI indexes were partially recovered (*P*<0.05). In the treatment group at a dose of 1000 mg/kg of body weight, the HOMA-IR index in the model group was statistically higher than that of the control and the treatment groups (*P*<0.05). HOMA-β, QUICKI, and DI indexes were also recovered (*P*<0.001 and *P*<0.05, respectively). The obtained results indicated that the EtOAc extract improved insulin sensitivity in diabetic mice. 

***Histopathology analysis of pancreas and livers in the experimental groups***

The data in Table 4 show the percentage of pancreatic mass compared with body weight in the diabetic group was statistically smaller than the control and treatment groups (*P*<0.001 and *P*<0.05, respectively). The treatment group of 1000 mg/kg body weight had a higher percentage of pancreatic mass than the lower dose group (*P*>0.05). 

**Figure 4:**
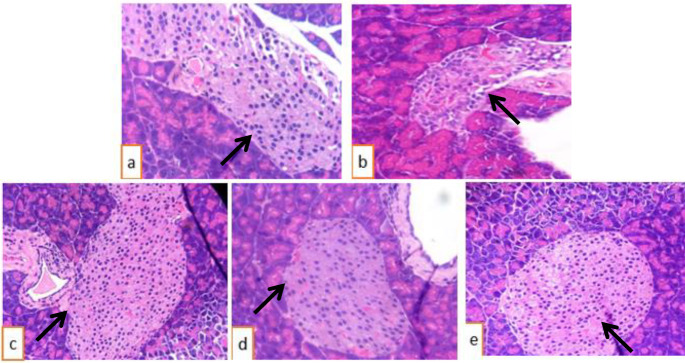
Hematoxylin and eosin stained histopathology of pancreas (x 400); (a) Control group; (b) Diabetic group without treatment; (c) Metformin group; (d) Treatment group (500 mg/kg of body weight); (e) Treatment group (1000 mg/kg of body weight)

Observation of the histopathology images (Figure 4) revealed pancreatic parenchyma with lobules separated by thin fibrous walls. The exocrine part of the pancreas in all control and treatment groups showed a normal appearance. The Langerhans islands (arrows) exhibited bright cytoplasmic cells. In the diabetic group, the Langerhans islands were smaller than those in the control and treatment groups. The quantitative data in Table 5 show that the diameters of the pancreatic islands were significantly smaller in the diabetic group compared with the control and treatment groups (*P*<0.05). Thus, the treatment groups had a reversed effect on pancreatic diameter compared with the control group (*P*>0.05), especially with the group of 1000 mg/kg of body weight (*P*>0.05). The results suggested that EtOAc extract helped to recover pancreatic structure in diabetic mice. 

**Figure 5:**
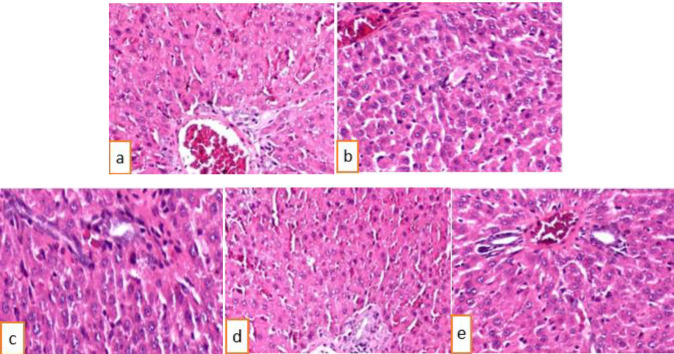
Hematoxylin and eosin stained histopathology of liver (x 400); (a) Control group; (b) Diabetic group without treatment; (c) Metformin group; (d) Treatment group (500 mg/kg of body weight); (e) Treatment group (1000 mg/kg of body weight)

The images of the liver under a microscope (DM 1000 Leica) (Figure 5) showed the structures of the liver cells in all groups were normal. There were no signs of inflammation, necrosis, or degeneration. Thus, EtOAc extract did not cause any adverse effect on mice livers.

**Table 1:** α-Glucosidase and α-amylase inhibitory activities of *Smilax glabra* ethyl acetate (EtOAc) extract. Data are presented as means±SD

Test samples	Activity IC_50_	
	AGI ( g/mL)	AAI (mg/mL)
EtOAc extract	1.15 0.03	0.13 0.02
Acarbose	359.21 5.06	0.09 0.03

AAI: α-amylase inhibitory; AGI: α-glucosidase inhibitory

**Table 2:** Effects of the ethyl acetate (EtOAc) extract on glucose and insulin levels in the blood (n=10)

Groups	Glucose (mg/dL)	Insulin (µIU/mL)
Control (1)	113.01 ± 4.24	15.47 ± 1.83
Diabetes (2)	195.46 ± 10.36	13.46 ± 1.43
Metformin (3)	115.60 ± 4.49	13.66 ± 1.61
Treatment (500 µg /kg) (4)	131.05 ± 5.32	13.60 ± 1.15
Treatment (1000 µg /kg) (5)	113.02 ± 4.00	13.82 ± 1.39
*P*	*P*_- 2_< 0.001; p_3,5- 1 _> 0.05; *P*_1,3,5- 4 _< 0.05; p_5- 3 _> 0.05;	*P*>0.05

P-2 : *P*-value compared with group 2 (model group)

P3,5- 1: *P*-value of group 3,5 compared with group 1 (control group)

P1,3,5- 4: *P*-value of groups 1,3,5 compared with group 4

P5- 3: *P*-value of groups 5 compared with group 3

**Table 3:** Effects of the ethyl acetate (EtOAc) extract on insulin resistance (n=10)

Group		HOMA-IR	HOMA-β	QUICKI	DI
Control	(1)	4.32 ± 0.54	121.25 ± 16.40	0.312 ± 0.005	29.70 ± 3.89
Diabetes	(2)	6.69 ± 0.96	37.48 ± 3.07	0.296 ± 0.005	6.44 ± 0.85
Metformin	(3)	3.91 ± 0.50	102.30 ± 14.23	0.317 ± 0.006	28.06 ± 4.27
Treatment (500 mg /kg)	(4)	4.37 ± 0.39	78.43 ± 9.53	0.310 ± 0.004	18.34 ± 2.11
Treatment (1000 mg /kg)	(5)	3.83 ± 0.38	108.77± 13.69	0.316 ± 0.005	29.35 ± 2.87
*P*		*P*_- 2_< 0.05; *P*_ 3,4,5- 1 _> 0.05; *P*_4,5- 3 _> 0.05; *P*_4-5 _> 0.05;	*P*_- 2_< 0.001; *P*_ ,5- 1 _> 0.05; *P*_4- 1 _< 0.05; * P*_4,5- 3 _> 0.05; 0.1> *P*_4-5 _> 0.05;	*P*_ - 2_< 0.05; * P*_ 3,4,5- 1 _> 0.05; *P*_ 4,5- 3 _> 0.05; * P*_ 4-5 _> 0.05;	*P*_- 2_< 0.001; *P*_3,5- 1 _> 0.05; _ 4-1 _< 0.05; *P*_4,5- 3 _> 0.05; *P*_4-5 _< 0.05;

HOMA: homeostatic model assessment; QUICKI: quantitative insulin sensitivity check index; DI: insulin disposition index

**Table 4:** The changes in pancreatic mass and diameter of pancreatic islets (n=10)

Groups		Pancreatic mass/body mass (%)
Control	(1)	0.927 ± 0.034
Diabetes	(2)	0.507 ± 0.024
Metformin	(3)	0.911 ± 0.046
Treatment (500 µg /kg)	(4)	0.881 ± 0.046
Treatment (1000 µg /kg)	(5)	0.948 ± 0.032
*P*		*P*_- 2_< 0.001; *P*_3.__,__4.__,__5- 1 _> 0.05; *P*_4__.__5- 3 _> 0.05; *P*_4-5 _> 0.05

**Table 5:** Changes in the diameter of pancreatic islets (n=10)

Groups		Diameter of pancreatic islets (µm)
Control	(1)	111.16 ± 5.99
Diabetes	(2)	81.84 ± 5.17
Metformin	(3)	105.32 ± 8.28
Treatment (500 µg /kg)	(4)	99.98 ± 5.80
Treatment (1000 µg /kg)	(5)	107.07 ± 7.09
*P*		*P*_-__2_< 0.05; *P*_3__.__4__.__5- 1 _> 0.05; *P*_4__.__5- 3 _> 0.05; *P*_4-5 _> 0.05

## Discussion

Our previous report (11) indicated that the EtOAc extract of *S. glabra *Roxb, a medicinal plant of Vietnam possessed a very high AGI compared with the reference compound acarbose (1 µg/ml vs 525 µg/ml) and significantly higher than the single active compounds astilbin and 5-*O*-caffeoylshikimic acid in the extract. Therefore, the EtOAc extract was chosen in this study to confirm its antidiabetic activity.

Dao *et al.* (18) and Nguyen *et al.* (19) reported that EtOH extract of *S. glabra *showed a hypoglycemic effect on type 1 *in vivo* models (STZ - induced diabetic mice, one of the animal models of insulin-dependent diabetes mellitus with hypoinsulinemia). Blood glucose level was also reported to be reduced by 26.78% in alloxan-induced diabetic mice after 7 days of oral administration of *S. glabra* EtOH extract. However, this activity has not been intensively investigated with the EtOAc extract in type 2 diabetic model. In this study, the EtOAc extract from *S. glabra*, for the first time, was investigated on acute toxicity and hypoglycemic activity in a mouse model for type 2 diabetes via the oral route. 

One of the typical features of type 2 diabetes is high glucose levels in the blood caused by the loss of insulin sensitivity or the increase in insulin resistance (IR) in the cells. In our study, we used STZ (low concentration) combined with NA to induce type 2 diabetes as previously reported (20-22). In this model, STZ partly causes pancreatic β-cell damage and DNA damage. In contrast, NA partially reduces the harmful effects of STZ via NO production, apoptosis prevention, and protection of the first phase of insulin release (14, 23, 24). Therefore, β-cells in these mice are only partially damaged, and insulin secretion is preserved but may not function well. Recently, using this model, betulinic acid (BA, a triterpene) was demonstrated to reduce blood glucose and α-amylase activity in plasma, to improve insulin sensitivity, as well as pancreatic histopathology (14). The same finding was reported with water and EtOH extracts of *Bromelia pumeiri* leaves on diabetic rats at a dose of 350 mg/kg of body weight (25). Thus, this model is suitable for our study. Because of bioavailability limitation, the plant extract is usually applied with high doses for diabetic treatment in *in vivo* models, for example, the doses of 800 and 1600 mg/kg of body weight of *Trichosanthes doica* extract were used for treatment of type 2 diabetic rats (26). In our study, the doses of 500 and 1000 µg/kg of body weight were selected based on our preliminary tests (data not shown) and the data reported for type 1 diabetic model by Dao *et al.* (18) and Nguyen *et al.* (19) using EtOH extract of *S. glabra*.

Interestingly, we found that the effects exerted by EtOAc extract on insulin biomarkers were similar to those associated with metformin treatment. Metformin is an anti-hyperglycemic agent, which improves glucose tolerance in patients with type 2 diabetes and lowers both basal and postprandial plasma glucose. Metformin also decreases hepatic glucose production, decreases intestinal absorption of glucose, and improves insulin sensitivity by increasing peripheral glucose uptake and utilization. This suggests that the mode of action of the EtOAc could be similar to metformin. Thus, the recovered pancreatic cells and restored insulin sensitivity are important mechanisms of the EtOAc extract to treat type 2 diabetes. These results agree well with findings by Funukaga *et al.* in terms of improvement of insulin sensitivity of *S. glabra* extrac (27). In this research, methanol extract of *S. glabra *showed hypoglycemic activity in both normal and KK-Ay transgenic mice with hyperinsulinemia at a dose of 100 µg/kg of body weight using intraperitoneal injection. KK-Ay mice showed significant decrease in blood glucose levels in an insulin tolerance test, demonstrating that the extract raised insulin sensitivity. A study found synergistic effects of hypoglycemic activity of the combination of metformin and oleanolic acid. Therefore, the combination of the EtOAc and the current commercial drugs, like metformin or acarbose, should be investigated to see if they have similar effects. 

It is known that enzyme inhibitors could be a potential target in disease control and treatment. An interesting study (20) showed that treatment with glycyrrhizinic acid (a triterpenoid of the ursane group) decreased HOMA-IR in diabetic mice due to a decrease in the gluconeogenesis eliminating enzymes. Another study (21) also isolated betulinic acid from *S. cumini* and found its effect on reduction of α-amylase activity in plasma. In our opinion, the high AGI and AAI activities of the EtOAc extract and its constituent compounds astilbin and 5-*O*-caffeoylshikimic acid could relate to the decrease of HOMA-IR and hypoglycemic activity. Further work should be carried out to clarify this hypothesis. 

Our evaluations of pancreatic weight changes (% compared with body weight) and histopathological analysis clearly indicated that the pancreatic islets recovered in size in treatment groups compared with the STZ-induced diabetic mice group. STZ is reported to have oxidative damage effects (14). Here, EtOAc extract seems to exhibit a reversible effect on oxidative damaged pancreatic cells. This result may relate to the antioxidant activity of the compound astilbin in the EtOAc extract (29), which could reduce oxidative damage by STZ (14). The same findings were also found for the compounds glycyrrhizic acid (30) and ursolic acid (31). How astilbin can reduce oxidative damage in the EtOAc extract-treated mice is still an unanswered question that requires further investigations.

## Conclusion

This is the first study to demonstrate the EtOAc extract of *S. glabra* has good hypoglycemic activity in the type 2 diabetic mouse model via oral route by lowering blood glucose levels and increasing insulin sensitivity in the treated mice. Although the EtOAc extract demonstrated potential effects, further evaluations on long-term administration of the extract in different *in vivo* models, including transgenic mice are needed to fully understand the mechanisms of action of this extract in diabetes control. Moreover, further clinical and chronic toxicity studies are required to confirm the beneficial effects of this extract for therapeutic applications.
